# New kidneys, old risks: cardiovascular challenges after transplantation

**DOI:** 10.1093/ndt/gfaf239

**Published:** 2025-11-06

**Authors:** Amaryllis H Van Craenenbroeck, Shanmugakumar Chinnappa, Evangelia Dounousi, Beatriz Fernandez-Fernandez, Fotini Iatridi, Patrick B Mark, Nejc Piko, Johannes Stegbauer, Claudia Torino, Liffert Vogt, Jose Manuel Valdivielso

**Affiliations:** Department of Nephrology, University Hospitals Leuven, Leuven, Belgium; Department of Microbiology, Immunology and Transplantation, Nephrology and Renal Transplantation Research Group, KU Leuven, Leuven, Belgium; Department of Nephrology, Doncaster and Bassetlaw Teaching Hospitals NHS Trust, Doncaster, UK; Leeds Institute of Cardiovascular and Metabolic Medicine (LICAMM), University of Leeds, Leeds, UK; Nephrology Department, School of Health Sciences, University of Ioannina and University Hospital of Ioannina, Ioannina, Greece; 2IIS-Fundacion Jimenez Diaz UAM, Madrid, Spain; Department of Medicine, School of Medicine, Universidad Autónoma de Madrid, Madrid, Spain; RICORS2040, Madrid, Spain; First Department of Nephrology, Hippokration Hospital, Aristotle University of Thessaloniki, Thessaloniki, Greece; School of Cardiovascular and Metabolic Health, University of Glasgow, Glasgow, UK; Renal and Transplant Unit, Queen Elizabeth University Hospital, Glasgow, UK; Department of Dialysis, Clinic for Internal Medicine, University Medical Centre Maribor, Maribor, Slovenia; Department of Nephrology, Medical Faculty, University Hospital Düsseldorf, Heinrich-Heine-University Düsseldorf, Düsseldorf, Germany; Cardiovascular Research Institute Düsseldorf, Medical Faculty, Heinrich Heine University, Germany; Institute of Clinical Physiology, National Research Council, Reggio Calabria, Italy; Department of Internal Medicine, Section of Nephrology, Amsterdam, The Netherlands; Amsterdam Cardiovascular Sciences, Amsterdam, The Netherlands; Vascular and Renal Translational Research Group, Institut de Recerca Biomedica de Lleida-Fundacion Dr Pifarre, IRBLleida, Lleida, Spain; RICORS2040, Madrid, Spain

**Keywords:** cardiovascular disease, kidney transplantation, management strategies

## Abstract

Kidney transplantation markedly improves survival and quality of life in patients with kidney failure, yet cardiovascular (CV) disease remains the leading cause of morbidity and mortality in kidney transplant recipients (KTRs). This review outlines the complex interplay of traditional, transplant-specific and recipient- and donor-related risk factors that sustain a high CV burden post-transplantation. While kidney function restoration reduces uremic toxins and improves cardiometabolic parameters, new challenges arise from immunosuppressive therapies, persistent hypertension, post-transplant diabetes mellitus and chronic inflammation. Common CV complications include coronary artery disease, heart failure, valvular disease, peripheral artery disease and refractory hypertension. Risk stratification tools and guidelines often fail to account for transplant-specific variables, resulting in suboptimal management. Although some pharmacological strategies and careful antihypertensive regimens show promise, most evidence is extrapolated from non-transplant populations due to the lack of dedicated randomized controlled trials. Emerging therapies like sodium-glucose co-transporter 2 inhibitors, glucagon-like peptide-1 receptor agonists and non-steroidal mineralocorticoid receptor antagonists hold potential but require further validation in this population. Moreover, sex disparities persist in access to transplantation and in post-transplant outcomes, with men generally experiencing higher CV risk but women potentially facing greater relative mortality. The review underscores the urgent need for transplant-specific CV research, personalized therapeutic strategies including precision medicine and greater inclusion of women in research. Optimizing CV outcomes in KTRs will require multidisciplinary collaboration, rigorous evidence generation, and an integrated approach to risk prediction, prevention and treatment.

## INTRODUCTION

Kidney transplantation is the preferred treatment for kidney failure, offering superior survival and quality of life as compared with dialysis [1]. It restores kidney function, improves metabolic balance and reduces complications associated with long-term dialysis [2]. However, despite these benefits, cardiovascular (CV) disease remains the leading cause of morbidity and mortality among kidney transplant recipients (KTRs) [3]. While transplantation reduces some traditional risk factors associated with kidney failure, such as uremia and fluid overload, it introduces new ones, including immunosuppressive therapy and metabolic disturbances [4]. These factors, combined with pre-existing ones, contribute to the high residual CV disease burden post-transplant. The advent of novel cardio- and nephroprotective therapy, such as sodium-glucose co-transporter 2 inhibitors (SGLT2i), glucagon-like peptide-1 receptor agonists (GLP1RA) and non-steroidal mineralocorticoid receptor antagonists (nsMRA), heralded an era of renewed optimism in nephrology. However, to date, no major studies have investigated their effects specifically in the KTR population. This review explores the intricate relationship between kidney transplantation and CV disease, emphasizing the persistent CV risks, the mechanisms driving post-transplant CV disease and potential strategies for mitigating CV complications, including how new knowledge and therapies might apply to this vulnerable population. Understanding these challenges is crucial for optimizing long-term outcomes and improving the overall prognosis of KTRs.

## CARDIOVASCULAR RISK FACTORS AND PATHOPHYSIOLOGY IN KIDNEY TRANSPLANTATION

**Figure 1:**
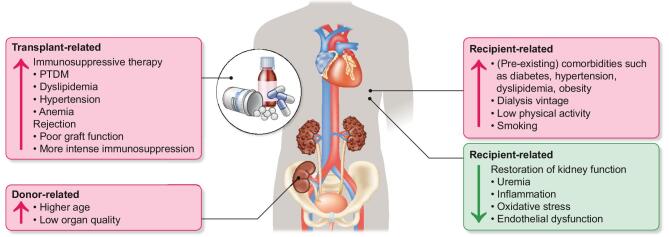
Cardiovascular risk factors after kidney transplantation. Three categories are present: recipient-, donor- and transplant-related factors. Red color represents an unfavorable risk profile, green an favorable risk profile.

Cardiovascular risk in KTRs can be broadly categorized into three groups: recipient-related CV risk factors, donor-related factors and transplant-related factors including the (adverse) effects of immunosuppressive therapy (see Fig. 1).

### Recipient-related factors

Kidney transplantation can substantially improve several CV risk factors that are prevalent in patients with kidney failure. For example, restoration of kidney function leads to the clearance of uremic toxins, known to promote endothelial dysfunction and inflammation, and to a better control of volume overload, which in turn alleviates cardiac stress. This improved hemodynamic balance has the potential to facilitate more effective blood pressure (BP) management, theoretically resulting in lower systolic and diastolic BP and ultimately contributing to enhanced CV stability. However, data show that, for example, the increased sympathetic activity observed in patients with kidney failure (one of the signals mediating the increase in BP) does not fully disappear after kidney transplantation unless native kidneys are removed, suggesting signals arising from native kidneys are independent of circulating uremia related toxins [5]. The recovery of kidney function improves inflammation and restores normal metabolic processes, which in turn supports the regulation of lipid metabolism. While in patients with advanced chronic kidney disease (CKD), the uremic milieu disrupts normal lipid processing with consequent dyslipidemia (characterized by elevated triglycerides and low-density lipoprotein cholesterol [6], disturbed lipoprotein particle size and number [7], and increasing atherosclerotic plaque formation [8]), in the post-transplant setting partial, but not full, normalization of these lipid profiles partially halting progression of atherosclerotic disease [9].

Parallel to these partial improvements after transplantation associated with recovery of kidney function, many traditional and kidney-specific CV risk factors may prevail in the recipient. Modifiable lifestyle choices include smoking and low physical activity, both important targets for intervention [10, 11]. Partially modifiable/treatable metabolic conditions such as diabetes, hypertension, dyslipidemia and obesity can be exacerbated after transplantation, in part due to the effects of immunosuppressive therapy. Higher dialysis vintage prior to kidney transplantation is also an independent factor for CV mortality post-transplant, with studies demonstrating a 10%–14% increase in CV death risk per year of pre-transplant dialysis [12]. Thus, timely preparation for pre-emptive transplantation or activation on the waiting list is key, and includes an assessment of current CV risk or prediction of the likelihood of major adverse CV events post-transplantation. Pretransplant CV evaluation is typically conducted to detect asymptomatic cardiac disease and to enable timely intervention aimed at improving both patient and allograft survival. Current clinical guidelines, however, rely heavily on expert opinion [3, 13, 14], and recent studies advocate for a more conservative screening approach, including the avoidance of preemptive revascularization in asymptomatic individuals [15–17].

### Donor-related factors

Donor-related factors may indirectly contribute to CV risk after kidney transplantation, primarily through their effect on graft function and inflammation. Higher donor age is associated not only with a greater incidence of delayed graft function (a pro-inflammatory state associated with increased CV events) but also chronic graft dysfunction, CKD and proteinuria—both recognized risk factors for CV complications [8, 18]. Similarly, suboptimal quality kidneys—including those with a history of hypertension or diabetes—have been linked to suboptimal graft outcomes and subsequently higher CV morbidity in recipients [19].

### Transplant-related factors

Specific transplant-related risk factors can offset the potential benefits of restored kidney function. Immunosuppressive regimens can cause or worsen dyslipidemia [20], hypertension [21], anemia [22] and diabetes [23], each of which may in turn accelerate the progression of CV disease. Post-transplant diabetes mellitus (PTDM) [24] and higher need for immunosuppression following rejection episodes significantly contribute to the increased CV risk of KTRs [25, 26]. We will discuss in detail all these factors in the following sections of this review.

## CARDIOVASCULAR COMPLICATIONS POST-TRANSPLANTATION

The key studies assessing CV disease in KTRs are summarized in Table 1. The most frequent CV complications after kidney transplantation are coronary artery disease (CAD), heart failure, valvular disease, peripheral artery disease (PAD) and hypertension.

**Table 1:** Key studies assessing CV disease in KTRs.

Type of study	Study population	Primary outcome	Secondary outcome	Results	Ref.
Cross-sectional	50 KTR	Extent of CAD in KT vs HD	Success of PCI in KT vs HD	Mean SYNTAX score: 13.3 ± 12.0 in KT, 20.6 ± 17.5 in HD and 9.4 ± 9.2 in control (*P* < .01). PCI successful in 93.8% of KTR, 75% of patients on HD, and 100% of control (*P* = .04)	[27]
Cross-sectional	79 KTR	Extent and characteristics of CAC at the time of KT		65% with CAC, median CAC scores higher in subjects with diabetes (127.8 vs 28.9; *P* = .05), exposed to dialysis (102.9 vs 3.7; *P* < .001) and deceased donor recipients (169.7 vs 7.5; *P* = .02)	[28]
Prospective cohort	67 KTR	CAC progression and 0 and 5 years after KT		At T0, 69% of patients with CAC and at T5, 76% with CAC. Progression of CAC in 74% of patients	[29]
Prospective registry analysis	2565 KTR and 25 650 controls	1- and 5-year incidence of CV events, kidney failure, and mortality following KT		1-year cumulative incidence of MI, stroke, or HF was 2.6% (95% CI 1.9%–3.2%) among KTR vs 0.5% (0.4%–0.5%) in controls. 5-year risk estimates for the same endpoints were 8.3% (7.1%–9.5%) for KTR and 2.6% (2.3%–2.8%) among controls. In KTR, cumulative mortality was 2.2% (1.7%–2.8%) and 10.3% (9.0%–11.6%) at 1 and 5 years, vs 0.5% (0.4%–0.6%) and 3.0% (2.7%–3.2%) for controls. The incidence of dialysis and re-transplantation was 6.1% (5.2%–7.1%) at 1 year and 16.3% (14.7%–17.9%) at 5 years	[33]
Prospective cohort	103 KTR with LVEF ≤40% and HF	Impact of KT on LVEF		Pre-transplant LVEF increased from 31.6 ± 6.7 (95% CI 30.3–32.9) to 52.2 ± 12.0 (95% CI 49.9–54.6; *P* = .002) at 12 months after KT	[35]
Retrospective registry analysis	27 011 KTR	Incidence of *de novo* HF after KT compared with patients on the waiting list		*De novo* HF incidence 10.2% (95% CI 9.8–10.6) and 18.3% (95% CI 17.8–18.9) at 12 and 36 months and decreased to less than the demographic-adjusted incidence on the waiting list	[37]
Retrospective registry analysis	35 215 KTR	Valvular heart disease after KT compared with HD		0.7/1000 patient years in KT recipients vs 2.2/1000 patients on HD (HR 0.28, 95% CI 0.17–0.47)	[38]
Retrospective registry analysis	19 329 adult KTR with diabetes	Impact of pre-transplant CAD and PAD on graft outcomes		Preexisting CAD and/or PAD result in worse posttransplant survival and CV outcomes	[42]
Retrospective cohort	819 KTR	Correlation between ABI, graft failure and mortality	Correlation between ABI and non-fatal MI, stroke, gangrene or limb amputation	Low ABI was a predictor of organ failure (OR 2.77, 95% CI 1.68–4.58; *P* < .001), secondary endpoints (HR 1.39, 95% CI 0.97–1.99; *P* < .076), and death (HR 1.84, 95% CI 1.26–2.68; *P* = .002)	[45]
Retrospective cohort	1666 KTR	Prevalence, treatment, control and clinical correlates of hypertension		sBP associated with relative risk for graft failure (1.12, 95% CI 1.08–1.15; *P* < .0001), death-censored graft failure (1.17, 1.12–1.22; *P* < .0001), and death (1.18, 1.12–1.23; *P* < .0001)	[47]
Prospective cohort	29 751 KTR	Role of BP on long-term kidney graft outcome		Increased levels of sBP and dBP post-transplantation were associated with a graded increase of subsequent graft failure (*P* < .0001)	[49]

ABI, ankle-brachial index; CAC, coronary artery calcification; CAD, coronary artery disease; CI, confidence interval; dBP, diastolic BP; HD, hemodialysis; HF, heart failure; HR, hazard ratio; KT, kidney transplantation; LVEF, left ventricular ejection fraction; MI, myocardial infarction; PAD, peripheral artery disease; PCI, percutaneous coronary intervention; sBP, systolic BP; SYNTAX, Synergy between percutaneous coronary intervention with TAXus and cardiac surgery.

Between 65% and 75% of KTR have coronary calcifications: this proportion is lower than the one observed in dialysis patients (92.1%) but higher than that observed in healthy controls (15.8%). Percutaneous cardiac intervention (PCI) success is highest in controls (100%), then in KTRs (93.8%), and lowest in dialysis patients (75%) [27, 28]. A rapidly progressing coronary artery calcification phenotype has been observed in up to 76% of KTRs 5 years after KT [29, 30], which is five times higher compared with the general population without any risk factors [31]. These findings underscore the persistent burden of CAD post-transplant, contributing to an annual myocardial infarction (MI) incidence of 1.5–2.6% and a 1-year mortality rate of 25% following acute coronary syndrome [32–34].

While left ventricular ejection fraction (LVEF) and left ventricle mass index may improve after kidney transplantation [35], heart failure and left ventricle hypertrophy (LVH) remain major contributors to CV-related hospitalizations [36]. *De novo* heart failure occurs in 10%–18% of KT patients within 12–36 months, increasing graft loss and mortality risks [37].

Valvular disease is common in KTRs, driven by CKD-related calcification and volume overload. Risk of hospitalizations may be reduced after kidney transplantation, however, patients requiring valve replacement (most commonly aortic or mitral) after kidney transplantation face a high mortality rate (∼20% per year) compared with controls [38]. Transcatheter aortic valve replacement seems to be at least comparable, if not superior to surgical technique for KTRs with severe aortic stenosis [39, 40], although studies with a higher number of patients are warranted. In comparison with hemodialysis patients, the rate of infectious complications of this technique is higher [41], but again, larger studies are needed to confirm this observation.

Beyond CAD and valvular disease, PAD significantly impacts post-transplant outcomes, particularly among diabetic patients, affecting up to 20% of them [42]. Registry data from the USA highlight preexisting CAD and PAD as major predictors of worse post-transplant survival and CV outcomes [43]. The incidence of intermittent claudication, revascularization (surgical/endovascular) or major amputation is 4.2% and 5.9% at 5- and 10-year follow-ups, respectively [44]. Additionally, an ankle-brachial index <0.9 or >1.4 after kidney transplantation is associated with an increased risk of graft loss and mortality at 5 years [45].

Another critical concern is post-transplant hypertension, affecting 80%–90% of KTRs [46]. A 5-year follow-up study of 1666 KTRs showed that only 4% had normal BP at 1 year without medication [47]. Hypertension post-transplant is closely linked to LVH, heart failure (particularly with preserved LVEF), and worsened allograft and CV outcomes [48, 49]. Effective BP management remains crucial for improving long-term transplant and CV outcomes, particularly considering that the rate of controlled BP in this population is often low compared with other CV risk populations [50]. The selection of antihypertensive therapy in kidney transplant recipients must consider both transplant-related and immunological factors. In cases of resistant hypertension, the prevalence of which is higher compared with the general population [51] but lower than in dialysis patients [52], management strategies may include targeted interventions such as transplant renal artery angioplasty with or without stenting, bilateral native nephrectomy or native kidney renal denervation, depending on individual patient characteristics [53].

### Sex disparities in cardiovascular outcomes

Sex disparities in KTRs begin before transplantation and persist post-transplant. Women donate kidneys more frequently but are less likely to receive adequate transplant counseling, be waitlisted or receive a living/deceased donor kidney [54]. A retrospective study of 2904 KTRs found that women had a lower risk of major adverse cardiac events (MACE) and nonfatal MI compared with men [55]. Similarly, among 30 325 KTRs in England, men faced a 20% higher risk than women for non-fatal MACE [56]. However, data from Australia and New Zealand suggest that women had a higher MACE risk post-transplant, particularly when the donor was male [57]. Nevertheless, women still remain underrepresented in kidney transplant clinical trials, comprising only ∼33% of study populations [58]. To ensure equitable, evidence-based care, clinical trials must include both sexes equally to generate definitive evidence applicable to all KTRs.

## WHAT LARGE DATASETS SHOW: MITIGATING CARDIOVASCULAR RISK IN KIDNEY TRANSPLANT RECIPIENTS

Although kidney transplantation is widely considered superior in improving both overall and CV survival, definitive evidence from randomized controlled trials (RCTs) is lacking, as it would be unethical to withhold a potentially life-saving treatment from eligible patients. Consequently, existing evidence relies heavily on observational studies, which, while informative, are inherently subject to selection bias and confounding. This is particularly relevant because candidates selected for transplantation often differ systematically from those who remain on dialysis. Nevertheless, the study by Sørensen *et al*. demonstrated a clear survival benefit of transplantation even in patients with high comorbidity, highlighting the potential advantages of the procedure despite methodological limitations [59]. To overcome these limitations, target trial emulation has emerged as a promising alternative, allowing for more robust causal inference without violating ethical principles. Preliminary results from a recent international target trial emulation presented at the ERA Congress 2025 suggest that this method may for instance help clarify the margins of survival benefit in deceased donor kidney transplantation, thus offering a more rigorous framework for future research [60].

Although post-transplant mortality and CV event rates have declined in recent years, their absolute incidence in KTRs remains substantial as outlined in the previous paragraphs [61]. A population study in Ontario involving 5248 recipients found a lower risk of MACEs and mortality in non-diabetic versus diabetic pre-transplant recipients (–50% MACE, –49% mortality 2 years after transplant). Similar results were observed comparing non-diabetics with recipients developing diabetes after transplant [62]. In a cohort of 43 006 KTRs from the Collaborative Transplant Study, high levels of systolic BP and pulse pressure were associated with all-cause mortality independently of the recipient’s age [63]. Similarly, in a retrospective observational study using the National Health Insurance Database of Korea (>14 000 KRTs) post-transplant hypertensive patients requiring multiple drugs showed a significantly higher risk of death without graft failure and MACEs [64]. A US Renal Data System-based study of 19 329 patients showed that CAD and/or PAD increased mortality risk by 40%–50%, with CAD also increasing the risk of MI [42]. Statin use was linked to reduced mortality in a real-life study involving 58 264 KTRs. The reduction in mortality was higher in those treated with calcineurin inhibitors (CNIs) (–38%), in patients with concomitant use of mammalian target of rapamycin kinase (mTOR) inhibitors (–27%) or non-treated with mycophenolate mofetil (–24%) [65].

Despite the insights gained about the residual CV risk after transplantation from observational studies, they again remain limited in their ability to infer causality. Therefore, better designed studies—such as the earlier mentioned target trial emulation and *in silico* clinical trials—are needed to identify modifiable CV risk factors and to determine whether modifying these factors translates into improved survival and CV outcomes. While numerous RCTs have addressed immunosuppression to optimize graft function and reduce risk of rejection, very few have prioritized CV outcomes as primary outcome in KTRs. It is now over 20 years since the ALERT (Assessment of Lescol in Renal Transplantation) trial and its subsequent extension study, which demonstrated in 2012 KTRs that lipid lowering with fluvastatin significantly reduced cardiac deaths and not fatal MI compared with placebo [66, 67]. The only other large CV outcome trial in KTRs, FAVORIT (Folic Acid for Vascular Outcome Reduction in Transplantation), showed no benefit of a combination of folic acid, vitamin B6 and vitamin B12 on CV events compared with placebo in 4110 KTRs [68]. It is disappointing that more CV outcome RCTs have not been performed in KTRs despite their high CV risk. Instead, we are obliged to extrapolate the results of trials such as those with sodium-glucose co-transporter 2 (SGLT2) inhibitors and glucagon-like peptide-1 receptor agonists (GLP-1RA) in the non-transplant CKD population with uncertainty around whether this is appropriate.

In addition to knowledge gained around the effect of an intervention on the primary endpoint, *post hoc* analyses of RCTs have been informative for better understanding risk factors for CV events in KTRs. Analysis of ALERT have shown that baseline CV disease and elevated cholesterol are risk factors for MI in KTRs, whereas diabetes, ischemic findings in electrocardiogram, graft function and age were risk factors for cardiac death [69]. Similar *post hoc* analyses of FAVORIT have demonstrated that CV risk factors are extremely common with suboptimal BP control in 44% of patients and that higher systolic BP was associated with CV events and CV death [70, 71].

It is well established that different immunosuppressive agents have varying effect on CV risk factors, e.g. CNIs are associated with salt-sensitive hypertension, tacrolimus and corticosteroids increase risk of post-transplant diabetes mellitus and mTOR inhibitors lead to elevation in cholesterol [72]. Although modification of the immunosuppressive regime to ameliorate CV risk profile is an attractive concept, trials of minimization of CNI using belatacept or mTOR inhibitors have not been associated with any demonstrable benefit in CV risk factor profile in KTRs [73, 74]. Another potential strategy to address CV risk is ligation of the arteriovenous fistula to improve LVH. This approach showed promising results in a pilot RCT, but a larger outcome trial will be required to confirm potential long term CV benefits [75].

## MANAGEMENT STRATEGIES

Implementing preventive and therapeutic strategies, particularly lifestyle modifications, remains crucial for reducing CV risk in this population—not only because kidney failure poses patients at an extremely high CV risk, but also because anti-rejection treatments influence the CV risk after transplantation [76]. For example, corticosteroid therapy may lead to a metabolic syndrome phenotype, including increased body mass, dyslipidemia, insulin resistance, hypertension, salt and fluid retention, and CNI use is associated with salt-sensitive hypertension, beta-cell destruction and diabetes, along with vasculopathy. Therefore, tailoring immunosuppression to reduce CV risk in KTRs is a critical strategy to improve long-term outcomes. The primary goal is to minimize corticosteroids and CNI without risking graft rejection. Personalized medicine using specific scores or donor-derived DNA levels to find and monitor the optimal immunosuppressive dose [77, 78] could be a promising future strategy [77, 78]. Although long-term data showed no beneficial effect on CV events, switching from a CNI-based to a belatacept-based regimen could reduce CV risk by improving glucose homeostasis and BP in KTRs with PTDM or difficult-to-treat hypertension [74, 79].

Pharmacological therapies suggested by various guidelines to control CV classical risk factors, hypertension, diabetes and dyslipidemia in KTRs, are summarized in Table 2.

**Table 2:** Guidelines—consensus on the pharmacological management of hypertension, diabetes mellitus and dyslipidemias in adult KTRs.

	Hypertension
KDIGO 2021 [128]	BP target <130/80 mmHg; standardized office measurements; first-line anti-hypertensive agent: dihydropiridine CCB or ARB (1C); if proteinuria, ARBs should be considered first, given their known antiproteinuric effect; avoid ARB in the early post-transplant period—until kidney function is stabilized
KDOQI 2021 (commentary on KDIGO 2021) [129]	BP target <130/80 mmHg; reasonable to treat with CCB on the basis of improved GFR and kidney survival; generally, in agreement with 2021 KDIGO; additional points: avoid non-dihydropyridine CCBs (e.g. diltiazem, verapamil) due to CYP3A4 inhibition; diuretics are useful for volume overload in KTRs but may increase AKI risk when combined with CNI; β-blockers are reserved for patients with comorbid coronary artery disease or heart failure
European Society Hypertension 2023 (endorsed by ISH and ERA) [130]	Office BP target <130/80 mmHg; first-line treatment dihydropyridine CCBs which may counteract CNI-induced vasoconstriction. Adjuvant therapies: RAS blockers (ACEis/ARBs), thiazide diuretics to block the cyclosporine-mediated sodium retention. For additional treatment option see antihypertensive therapy in CKD
European Society of Cardiology 2024 [131]	Individualized BP targets in KTRs based on tolerability and renal function; not specific recommendations about anti-hypertensive drug category
UK Renal Association and British Transplantation Society 2017 [132]	Clinic BP target <140/90 mmHg (2C); target BP 130/80 mmHg if UPCR >50 mg/mmol or UACR >35 mg/mmol (2C); no evidence that any antihypertensive agent is better than any other, and effort should be focused on achieving absolute BP levels rather than preferring specific individual agents (2D); RAS inhibitors may be more effective in the minimization of proteinuria but use with caution in the first 3 months post-transplant (2C)
American Heart Association 2017 [133]	BP target <130/80 mmHg; reasonable to treat with CCB on the basis of improved GFR and kidney survival; does not mention anti-RAS
	**Diabetes mellitus**
UK Renal Association and British Transplantation Society 2017 [132]	No recommendation for specific drug category; post-transplant diabetes should be managed in collaboration with specialists in diabetic medicine (2D); all units should have a protocol for the management of PTDM (2C)
KDIGO 2022 [134, 135]	Comprehensive care: lifestyle interventions; metformin recommended if eGFR ≥30 mL/min/1.73 m^2^, long acting GLP-1RA recommended if glycemic targets not achieved despite use of metformin, or unable to use; in KTRs with diabetes, hypertension and albuminuria, initiate ACEi or ARB and titrate to the highest approved dose that is tolerated; SGLT2 inhibitors not recommended
KDOQI 2022 (commentary on KDIGO 2020) [136]	Agreement with KDIGO recommendations; only modification: “long acting GLP-1RA with proven cardiovascular benefits”
American Diabetes Association 2025 [137]	Insulin therapy immediately posttransplant and noninsulin therapies for long-term management; metformin recommended up to eGFR ≥30 mL/min/1.73 m^2^; GLP-1RA recommended; SGLT2 inhibitors may be preferred for diabetic KTRs with ASCVD, HF and CKD but with increased risk of genitourinary tract infection
International consensus 2024 [93]	Early insulin initiation common; use of DPP4 inhibitors/metformin early post-transplant; later use of SGLT2 inhibitors and GLP-1RA; immunosuppressive regimen adjustments to reduce PTDM risk
	**Dyslipidemias**
KDIGO 2013 [138]	Statin therapy suggested (2B); no specific LDL-C target defined; PCSK-9 not mentioned; lifestyle changes for hypertriglyceridemia
KDOQI 2015 (commentary on KDIGO 2013) [139]	Endorses KDIGO 2013 recommendations
UK Renal Association and British Transplantation Society 2017 [132]	Treatment targets for dyslipidemia should be the same as in the general population (2C); KTRs at increased primary or secondary CV risk should receive statin (2C); choice of statin and dose should reflect concurrent immunosuppression, high-dose simvastatin (≥40 mg/day) should be avoided in conjunction with ciclosporin and CCB (2D)
European Society of Cardiology 2019 [140]	Statins should be considered as first-line agents, initiation should be at low doses with careful uptitration and with caution regarding potential drug-to-drug interactions, particularly for patients on ciclosporin (IIa-B); in patients who are intolerant of statins or those with significant dyslipidemia despite maximally tolerated statin treatment, alternative or additional therapy with ezetimibe may be considered (IIb-C); care is required with the use of fibrates, as they can decrease ciclosporin levels and have the potential to cause myopathy; extreme caution is required if fibrate therapy is planned in combination with a statin; management of dyslipidemias in KTRs is comparable to what is recommended for patients at high or very high ASCVD risk

AKI, acute kidney injury; ACEi, angiotensin-converting enzyme inhibitor; ARB, angiotensin II receptor blocker; ASCVD, atherosclerotic CV disease; DPP4, dipeptidyl peptidase-4; ISH, International Society of Hypertension; LDL-C, low-density lipoprotein cholesterol; PCSK9, proprotein convertase subtilisin/kexin type 9; RAS, renin-angiotensin system; SGLT2i, SGLT2 inhibitor; UACR, urine albumin:creatinine ratio; UPCR, urine protein:creatinine ratio.

Due to a lack of large RCTs in KTRs, the level of recommendation is in general quite low. In summary, for hypertension in KTRs, most guidelines agree to a BP target <130/80 mmHg. As first-line treatment, most guidelines recommend dihydropyridine calcium channel blockers (CCBs) in combination with renin–angiotensin system inhibitors, mainly angiotensin receptor blockers, when transplant function is stabilized or proteinuria occurred. Of note, as non-dihydropyridine CCBs are CYP3A4 inhibitors, specific attention should be paid to CNI concentrations when coadministration is unavoidable. Adjunctive therapies with thiazides or loop diuretics, β-blockers, α-blockers and MRAs are suggested, depending on indication. For the management of diabetes in KTRs, insulin therapy immediately after transplantation and noninsulin therapies for long-term management are suggested. In particular, metformin is recommended for estimated glomerular filtration rate (eGFR) ≥30 mL/min/1.73 m^2^ and GLP-1RA with CV benefit if glycemic targets are not achieved despite the use of metformin. The recommendation for metformin is derived from CKD guidelines in patients with diabetes [80], but its direct extrapolation to transplant recipients may not always be appropriate, given their less stable kidney function and higher risk of acute deterioration during infectious episodes, nephrotoxic drugs, or rejection. Thus, when using metformin, eGFR should be monitored regularly and dose reduction or introduction of a “sick day” rule should be considered when eGFR drops below <60 mL/min/1.73 m^2^ or in the presence of predisposing factors for hypoperfusion and hypoxemia. SGLT2 inhibitors may be used in diabetic KTRs with atherosclerotic CV disease, heart failure or CKD. Due to the lack of RCTs, no clear agreement between guidelines exists, but data on the favorable safety profile of SGLT2 inhibition is accumulating [81–83]. KTRs at increased primary or secondary CV risk should receive statins (fluvastatin or atorvastatin) as first-line agents, based primarily on the ALERT trial for fluvastatin and extensive pharmacokinetic data [67]. In general, simvastatin is not preferred due to a perceived higher risk of rhabdomyolysis, particularly in combination with ciclosporin [84]. Initiation should be at low doses with careful up-titration and with caution regarding potential drug-to-drug interactions, particularly with ciclosporin, although the widespread use of tacrolimus as CNI has lessened this issue [84]. Alternative or additional therapy with ezetimibe [85] may be considered as this agent appears safe, although it lacks hard outcome trials, while the use of fibrates is discouraged [86]. Of note, evidence on proprotein convertase subtilisin-kexin type 9 (PSCK9) inhibition is scarce but promising [87].

Newer pharmacological agents with proven renoprotection and cardioprotection in native kidneys, are not generally recommended for KTRs, due to lack of evidence. In particular, landmark SGLT2 inhibitor studies have excluded KTRs and available data remain limited [88–92]. The main concerns about the safety of SGLT2 inhibitors in KTRs include increased risk of urinary tract infections and the potential risk of hypotension due to osmotic diuresis and acute drops in the eGFR, although reassuring results have been shown in recent studies [81–83]. The recent 2025 consensus on PTDM states that SGLT2 inhibitors can be used for the treatment of PTDM once stable graft function is achieved considering CV comorbidities and advising patient for “sick days” rules [93]. Nevertheless, the extent to which SGLT2 inhibitors confer beneficial effects both in diabetic and non-diabetic KTRs remains an open question to be addressed by ongoing and future randomized studies. In the same line, there is a paucity of evidence on the utilization of steroidal and nonsteroidal MRAs in KTRs. Although preliminary data suggest advantageous effects of MRAs on patterns of injury such as ischemia–reperfusion injury or CNI-mediated nephrotoxicity, a study of spironolactone added to standard-of-care therapy did not yield any favorable graft outcomes [94, 95]. An ongoing multicenter, phase 2 randomized, double blinded, placebo controlled clinical trial will shed light on the safety and efficacy of finerenone in KTRs (“The EFfect of FinErenone in Kidney TransplantiOn Recipients: The EFFEKTOR Study,” NCT06059664). An exception are the glucagon-like peptide-1 receptor agonists (GLP1RA), which have shown to have benefits in terms of glycemic control and body weight reduction, without significant effects on BP control or renal function for diabetic KTRs (Table 2). Despite the potential kidney and CV protective effects of GLP1RA in diabetic KTRs, the increased likelihood for developing drug-related complications such as acute pancreatitis or medullary thyroid cancer are the main concerns [96, 97]. In this regard, well-designed future studies including larger populations and with a long follow-up are needed to clarify these pending issues, some of which are already being conducted: “The Efficacy, Mechanism & Safety of Sodium Glucose Co-Transporter-2 Inhibitor & Glucagon-Like Peptide 1 Receptor Agonist Combination Therapy in Kidney Transplant Recipients (HALLMARK),” NCT05938712; “Semaglutide Treatment for Hyperglycaemia After Renal Transplantation (Sema-RTx),” NCT05702931; and “Obesity Management for Kidney TRANSPLANTation: OK-TRANSPLANT 2,” NCT06396416.

Besides pharmacological treatment, lifestyle modifications remain of paramount importance in CV risk reduction. A diet low in sodium and saturated fat helps control hypertension and dyslipidemia, while a balanced intake of fruits, vegetables and whole grains supports overall CV health [76, 98–100]. Regular physical activity aids weight management, BP control and lipid balance, and exercise programs should be tailored to transplant recipients’ needs [101, 102]. Smoking cessation is an undiminished essential [98], also for potential donors [103]. Unfortunately, current practice data from various countries indicate that it is difficult to achieve these goals [104, 105]. Very active monitoring of lifestyle factors, alongside the complex care required to optimize immunosuppression and manage comorbidities (e.g. reduced bone density, infections, malignancies), is therefore persistently essential for achieving guideline-recommended CV outcomes [98, 106] and should be actively pursued by transplant caregivers.

## EMERGING THERAPIES AND RESEARCH PRIORITIES

Recent advancements in therapeutic strategies have demonstrated significant benefits in mitigating CV risks for KTRs [107]. Given the CV burden associated with traditional immunosuppressants, optimization of immunosuppressive therapy has become a key area of focus. Tacrolimus conversion to alternative regimens such as ciclosporin has been associated with an improved glucose metabolism [108]. In the HARMONY trial, rapid steroid withdrawal in patients with low immunological risk significantly reduced the incidence of PTDM [109]. Additionally, belatacept, a newer immunosuppressant mainly studied in patients with low immunological risk, has shown improved kidney function, better BP levels, lipid control and lower incidence of PTDM, although long-term data showed no beneficial effects on CV events [110, 111]. Newer anti-diabetic agents, particularly SGLT2 inhibitors and GLP-1RAs, have been associated with improved CV and kidney outcomes in patients with CKD independently of their diabetic status [112–115], while retrospective data suggest similar benefits in diabetic KTRs [90] highlighting their potential to improve CV risk in this population. Several ongoing studies currently examining effects of these agents specifically in KTRs are expected to shed light in this field: “CardioRenal Effects of SGLT2 Inhibition in Kidney Transplant Recipients (CREST-KT),” NCT04906213; “Efficacy, Mechanisms and Safety of SGLT2 Inhibitors in Kidney Transplant Recipients (INFINITI2019),” NCT04965935; “Can Dapagliflozin Preserve Structure and Function in Transplanted Kidneys? (DEAKTransplant),” NCT05788276; “The RENAL LIFECYCLE Trial: A RCT to Assess the Effect of Dapagliflozin on Renal and Cardiovascular Outcomes in Patients With Severe CKD,” NCT05374291; and “Semaglutide Treatment for Hyperglycaemia After Renal Transplantation (Sema-RTx),” NCT05702931.

Precision medicine and sex-specific approaches have gained prominence in CV disease management. Among KTRs, men seem to have a higher risk of major CV events than women [56]; however, evidence from large cohorts suggest that women exhibit higher excess of mortality risk than men, as compared with the general population [58, 116]. Structural cardiac changes also exhibit sex-specific trends, with women displaying a greater propensity for LVH 1 year post-transplantation [117]. These disparities may be driven by hormonal differences, variations in BP control and patterns of CV remodeling [118], while the increasingly recognized effects of genomic and epigenomic factors may also contribute [119]. Recent efforts in improving sex-specific CV risk prediction tools and genetic risk scores could help employ sex-specific approaches for KTRs and optimize post-transplant care [120, 121].

### Knowledge gaps

Whereas screening for CV disease in potential kidney transplant candidates remains topical and draws considerable research interest, CV risk assessment in KTRs remains a “Cinderella” topic. Traditional risk scores such as Framingham score underestimate CVD risk in KTRs [122]. An appropriate cardiac risk assessment tool in this cohort should include transplant-specific risk factors such as immunosuppression, graft function, chronic inflammation, etc., in addition to traditional risk factors. The Patient Outcomes in Renal Transplantation (PORT) study and CV Risk Calculator for Renal Transplant Recipients (CRCRTR-MACE) score are significant steps in this direction [123, 124]. In addition to development of better screening tools, further research is also required in prevention, early detection and treatment of CVD in KTRs. Research should focus on long-term outcomes of such therapies as the pathogenesis of CVD in KTRs runs an indolent course with a median time to CVD events of 5.8 years (interquartile range 2.5–6.2), as shown in a recent study [125].

As cardiology trials tend to exclude CKD patients [126], CKD-specific trials are essential to build strong evidence base for the management of CVD. Even when such trials are conducted, they tend to happen in silos such as early CKD, pre-dialysis CKD, dialysis or transplantation. As patients with CKD journey through these phases, they encounter various care teams, different priorities and conflicting recommendations even for the same medication. Hence cardio-kidney research requires a more joined-up thinking to take a more coherent direction. Notably, clinical trials in kidney transplantation have historically underrepresented women, limiting the availability of sex-specific data to guide management [58]. Addressing these gaps requires a rigorous effort for balanced sex representation. Additionally, the associations of sex with graft failure and mortality with functioning graft may be in opposite directions and should be analysed separately, while taking into account potential modifying effects of recipient and donor age [127]. Integrating precision medicine and sex-based associations into post-transplant CV management is essential to improve patient outcomes.

## CONCLUSION

In summary, while kidney transplantation is lifesaving, CV complications remain a major cause of illness and death. Ongoing risks like CAD, hypertension and PAD call for early detection and personalized management. Sex disparities underscore the need for inclusive research and care. Improving outcomes will require targeted strategies and multidisciplinary collaboration.

## Data Availability

Not applicable.

## References

[1] Wolfe RA, Ashby VB, Milford EL et al. Comparison of mortality in all patients on dialysis, patients on dialysis awaiting transplantation, and recipients of a first cadaveric transplant. N Engl J Med 1999;341:1725–30. 10.1056/NEJM199912023412303 10580071

[2] Tonelli M, Wiebe N, Knoll G et al. Systematic review: kidney transplantation compared with dialysis in clinically relevant outcomes. Am J Transplant 2011;11:2093–109. 10.1111/j.1600-6143.2011.03686.x 21883901

[3] Rangaswami J, Mathew RO, Parasuraman R et al. Cardiovascular disease in the kidney transplant recipient: epidemiology, diagnosis and management strategies. Nephrol Dial Transplant 2019;34:760–73. 10.1093/ndt/gfz053 30984976

[4] Phillips S, Heuberger R. Metabolic disorders following kidney transplantation. J Ren Nutr 2012;22:451–60.e1. 10.1053/j.jrn.2012.01.022 22445053

[5] Hausberg M, Kosch M, Harmelink P et al. Sympathetic nerve activity in end-stage renal disease. Circulation 2002;106:1974–9. 10.1161/01.CIR.0000034043.16664.96 12370222

[6] Betriu A, Martinez-Alonso M, Arcidiacono MV et al. Prevalence of subclinical atheromatosis and associated risk factors in chronic kidney disease: the NEFRONA study. Nephrol Dial Transplant 2014;29:1415–22. 10.1093/ndt/gfu038 24586070

[7] Bermudez-Lopez M, Forne C, Amigo N et al. An in-depth analysis shows a hidden atherogenic lipoprotein profile in non-diabetic chronic kidney disease patients. Expert Opin Ther Targets 2019;23:619–30. 10.1080/14728222.2019.1620206 31100024

[8] Valdivielso JM, Rodríguez-Puyol D, Pascual J et al. Atherosclerosis in chronic kidney disease: more, less, or just different? Arterioscler Thromb Vasc Biol 2019;39:1938–66. 10.1161/ATVBAHA.119.312705 31412740

[9] Meier-Kriesche H-U, Schold JD, Srinivas TR et al. Kidney transplantation halts cardiovascular disease progression in patients with end-stage renal disease. Am J Transplant 2004;4:1662–8. 10.1111/j.1600-6143.2004.00573.x 15367222

[10] Corbett C, Armstrong MJ, Neuberger J. Tobacco smoking and solid organ transplantation. Transplantation 2012;94:979–87. 10.1097/TP.0b013e318263ad5b 23169222

[11] Zelle DM, Klaassen G, van Adrichem E et al. Physical inactivity: a risk factor and target for intervention in renal care. Nat Rev Nephrol 2017;13:152–68. 10.1038/nrneph.2016.187 28138130

[12] Helanterä I, Salmela K, Kyllönen L et al. Pretransplant dialysis duration and risk of death after kidney transplantation in the current era. Transplantation 2014;98:458–64. 10.1097/TP.0000000000000085 24646770

[13] Cheng XS, VanWagner LB, Costa SP et al. Emerging evidence on coronary heart disease screening in kidney and liver transplantation candidates: a scientific statement from the American Heart Association: endorsed by the American Society of Transplantation. Circulation 2022;146:e299–324. 10.1161/CIR.0000000000001104 36252095 PMC10124159

[14] Chadban SJ, Ahn C, Axelrod DA et al. KDIGO Clinical Practice Guideline on the Evaluation and Management of Candidates for Kidney Transplantation. Transplantation 2020;104:S11–103. 10.1097/TP.0000000000003136 32301874

[15] Deak AT, Ionita F, Kirsch AH et al. Impact of cardiovascular risk stratification strategies in kidney transplantation over time. Nephrol Dial Transplant 2020;35:1810–8. 10.1093/ndt/gfaa131 33022711 PMC7538198

[16] Siddiqui MU, Junarta J, Marhefka GD. Coronary revascularization versus optimal medical therapy in renal transplant candidates with coronary artery disease: a systematic review and meta-analysis. J Am Heart Assoc 2022;11:e023548. 10.1161/JAHA.121.023548 35132876 PMC9245820

[17] Beaudrey T, Bedo D, Weschler C et al. From risk assessment to management: cardiovascular complications in pre- and post-kidney transplant recipients: a narrative review. Diagnostics 2025;15:802. 10.3390/diagnostics15070802 40218153 PMC11988545

[18] Oppenheimer F. The impact of donor age on the results of renal transplantation. Nephrol Dial Transplant 2004;19(Suppl 3):iii11–15. 10.1093/ndt/gfh1008 15192129

[19] Blanca L, Jiménez T, Cabello M et al. Cardiovascular risk in recipients with kidney transplants from expanded criteria donors. Transplant Proc 2012;44:2579–81. 10.1016/j.transproceed.2012.09.086 23146460

[20] Cosio FG, Pesavento TE, Pelletier RP et al. Patient survival after renal transplantation III: the effects of statins. Am J Kidney Dis 2002;40:638–43. 10.1053/ajkd.2002.34927 12200817

[21] Rubin MF. Hypertension following kidney transplantation. Adv Chronic Kidney Dis 2011;18:17–22. 10.1053/j.ackd.2010.10.006 21224026

[22] Mix TCH, Kazmi W, Khan S et al. Anemia: a continuing problem following kidney transplantation. Am J Transplant 2003;3:1426–33. 10.1046/j.1600-6135.2003.00224.x 14525605

[23] Cosio FG, Pesavento TE, Osei K et al. Post-transplant diabetes mellitus: increasing incidence in renal allograft recipients transplanted in recent years. Kidney Int 2001;59:732–7. 10.1046/j.1523-1755.2001.059002732.x 11168956

[24] Cosio FG, Kudva Y, van der Velde M et al. New onset hyperglycemia and diabetes are associated with increased cardiovascular risk after kidney transplantation. Kidney Int 2005;67:2415–21. 10.1111/j.1523-1755.2005.00349.x 15882287

[25] Okamoto T, Hatakeyama S, Hamaya T et al. Impact of timing of rejection episode on cardiovascular events in living donor kidney transplantation: a multicenter retrospective study. J Nephrol 2023;36:2613–20. 10.1007/s40620-023-01811-9 37938544

[26] Amornkanjanawat P, Kerr SJ, Wuttiputhanun T et al. Kidney allograft rejection as an independent non-traditional risk factor for post-transplant cardiovascular events. Kidney360 2025;6:1176–87. 10.34067/KID.0000000773 PMC12338360 40105892

[27] Paizis IA, Mantzouratou PD, Tzanis GS et al. Coronary artery disease in renal transplant recipients: an angiographic study. Hellenic J Cardiol 2020;61:199–203. 10.1016/j.hjc.2018.07.002 29981889

[28] Rosas SE, Mensah K, Weinstein RB et al. Coronary artery calcification in renal transplant recipients. Am J Transplant 2005;5:1942–7. 10.1111/j.1600-6143.2005.00955.x 15996243

[29] Alfieri C, Forzenigo L, Tripodi F et al. Long-term evaluation of coronary artery calcifications in kidney transplanted patients: a follow up of 5 years. Sci Rep 2019;9:6869. 10.1038/s41598-019-43216-4 31053792 PMC6499881

[30] Seyahi N, Alagoz S, Atli Z et al. Coronary artery calcification progression and long-term cardiovascular outcomes in renal transplant recipients: an analysis by the joint model. Clin Kidney J 2022;15:101–8. 10.1093/ckj/sfab174 35106150 PMC8796795

[31] Yoo J-Y, Kang S-R, Chun E-J. Progression of coronary artery calcification according to changes in risk factors in asymptomatic individuals. J Pers Med 2024;14:757. 10.3390/jpm14070757 39064011 PMC11278493

[32] Kasiske BL, Maclean JR, Snyder JJ. Acute myocardial infarction and kidney transplantation. J Am Soc Nephrol 2006;17:900–7. 10.1681/ASN.2005090984 16481414

[33] Andersson C, Hansen D, Sørensen SS et al. Long-term cardiovascular events, graft failure, and mortality in kidney transplant recipients. Eur J Intern Med 2024;121:109–13. 10.1016/j.ejim.2023.10.026 37903704

[34] Birdwell KA, Park M. Post-transplant cardiovascular disease. Clin J Am Soc Nephrol 2021;16:1878–89. 10.2215/CJN.00520121 34556500 PMC8729500

[35] Wali RK, Wang GS, Gottlieb SS et al. Effect of kidney transplantation on left ventricular systolic dysfunction and congestive heart failure in patients with end-stage renal disease. J Am Coll Cardiol 2005;45:1051–60. 10.1016/j.jacc.2004.11.061 15808763

[36] Tian Z, Bergmann K, Kaufeld J et al. Left ventricular hypertrophy after renal transplantation: systematic review and meta-analysis. Transplant Direct 2024;10:e1647. 10.1097/TXD.0000000000001647 38769973 PMC11104731

[37] Lentine KL, Schnitzler MA, Abbott KC et al. De novo congestive heart failure after kidney transplantation: a common condition with poor prognostic implications. Am J Kidney Dis 2005;46:720–33. 10.1053/j.ajkd.2005.06.019 16183428

[38] Abbott KC, Hshieh P, Cruess D et al. Hospitalized valvular heart disease in patients on renal transplant waiting list: incidence, clinical correlates and outcomes. Clin Nephrol 2003;59:79–87. 10.5414/CNP59079 12608550

[39] Mir T, Darmoch F, Ullah W et al. Transcatheter versus surgical aortic valve replacement in renal transplant patients: a meta-analysis. Cardiol Res 2020;11:280–5. 10.14740/cr1092 32849962 PMC7430886

[40] Fong KY, Ong JHW, Chan YH et al. A systematic review and meta-analysis of transcatheter versus surgical aortic valve replacement in kidney transplant patients. Am J Cardiol 2023;204:22–5. 10.1016/j.amjcard.2023.07.033 37536199

[41] Al-Rashid F, Bienholz A, Hildebrandt HA et al. Transfemoral transcatheter aortic valve implantation in patients with end-stage renal disease and kidney transplant recipients. Sci Rep 2017;7:14397. 10.1038/s41598-017-14486-7 29089579 PMC5663698

[42] Jiwani S, Chan W-C, Majmundar M et al. Impact of preexisting coronary artery and peripheral artery disease on outcomes in diabetic patients after kidney transplant. Vasc Med 2024;29:135–42. 10.1177/1358863X231205574 37936422

[43] Snyder JJ, Kasiske BL, Maclean R. Peripheral arterial disease and renal transplantation. J Am Soc Nephrol 2006;17:2056–68. 10.1681/ASN.2006030201 16775031

[44] Sung RS, Althoen M, Howell TA et al. Peripheral vascular occlusive disease in renal transplant recipients: risk factors and impact on kidney allograft survival. Transplantation 2000;70:1049–54. 10.1097/00007890-200010150-00010 11045641

[45] Patel SI, Chakkera HA, Wennberg PW et al. Peripheral arterial disease preoperatively may predict graft failure and mortality in kidney transplant recipients. Vasc Med 2017;22:225–30. 10.1177/1358863X16689830 28466760

[46] Weir MR, Burgess ED, Cooper JE et al. Assessment and management of hypertension in transplant patients. J Am Soc Nephrol 2015;26:1248–60. 10.1681/ASN.2014080834 25653099 PMC4446882

[47] Kasiske BL, Anjum S, Shah R et al. Hypertension after kidney transplantation. Am J Kidney Dis 2004;43:1071–81. 10.1053/j.ajkd.2004.03.013 15168388

[48] Yancy CW, Lopatin M, Stevenson LW et al. Clinical presentation, management, and in-hospital outcomes of patients admitted with acute decompensated heart failure with preserved systolic function: a report from the Acute Decompensated Heart Failure National Registry (ADHERE) Database. J Am Coll Cardiol 2006;47:76–84. 10.1016/j.jacc.2005.09.022 16386668

[49] Opelz G, Wujciak T, Ritz E. Association of chronic kidney graft failure with recipient blood pressure. Collaborative Transplant Study. Kidney Int 1998;53:217–22. 10.1046/j.1523-1755.1998.00744.x 9453022

[50] Pisano A, Mallamaci F, D’Arrigo G et al. Assessment of hypertension in kidney transplantation by ambulatory blood pressure monitoring: a systematic review and meta-analysis. Clin Kidney J 2022;15:31–42. 10.1093/ckj/sfab135 35035934 PMC8757429

[51] Arias-Rodríguez M, Fernández-Fresnedo G, Campistol JM et al. Prevalence and clinical characteristics of renal transplant patients with true resistant hypertension. J Hypertens 2015;33:1074–81. 10.1097/HJH.0000000000000510 25668343

[52] Georgianos PI, Agarwal R. Resistant hypertension in dialysis: epidemiology, diagnosis, and management. J Am Soc Nephrol 2024;35:505–14. 10.1681/ASN.0000000000000315 38227447 PMC11000742

[53] Tantisattamo E, Molnar MZ, Ho BT et al. Approach and management of hypertension after kidney transplantation. Front Med 2020;7:229. 10.3389/fmed.2020.00229 PMC7310511 32613001

[54] Nautiyal A, Bagchi S, Bansal SB. Gender and kidney transplantation. Front Nephrol 2024;4:1360856. 10.3389/fneph.2024.1360856 38711923 PMC11070561

[55] Liu J, Chen S, Gao W. Gender differences in cardiovascular outcomes of kidney transplant recipients: a retrospective cohort study. Medicine (Baltimore) 2024;103:e39568. 10.1097/MD.0000000000039568 39287307 PMC11404969

[56] Anderson B, Qasim M, Evison F et al. A population cohort analysis of English transplant centers indicates major adverse cardiovascular events after kidney transplantation. Kidney Int 2022;102:876–84. 10.1016/j.kint.2022.05.017 35716956

[57] Vinson AJ, Zhang X, Dahhou M et al. A multinational cohort study uncovered sex differences in excess mortality after kidney transplant. Kidney Int 2023;103:1131–43. 10.1016/j.kint.2023.01.022 36805451

[58] Vinson AJ, Ahmed SB. Representation of women in contemporary kidney transplant trials. Transpl Int 2023;36:11206. 10.3389/ti.2023.11206 37125385 PMC10141646

[59] Sørensen VR, Heaf J, Wehberg S et al. Survival benefit in renal transplantation despite high comorbidity. Transplantation 2016;100:2160–7. 26599492 10.1097/TP.0000000000001002 PMC5120769

[60] Hellemans R, Chesnaye N, Kramer A et al. Exploring the Margins of Survival Benefit in Deceased Donor Kidney Transplantation: an International Target Trial Emulation. Presented at the ERA Congress 2025, 4–7 June 2025, Vienna, Austria.

[61] Li Y, Menon G, Wu W et al. Evolving trends in kidney transplant outcomes among older adults: a comparative analysis before and during the COVID-19 pandemic. Transplant Direct 2023;9:e1520. 10.1097/TXD.0000000000001520 37928483 PMC10624464

[62] Lim WH, Lok CE, Kim SJ et al. Impact of pretransplant and new-onset diabetes after transplantation on the risk of major adverse cardiovascular events in kidney transplant recipients: a population-based cohort study. Transplantation 2021;105:2470–81. 10.1097/TP.0000000000003639 33560726

[63] Krüger B, Döhler B, Opelz G et al. Pulse pressure and outcome in kidney transplantation: results from the Collaborative Transplant Study. Transplantation 2019;103:772–80. 30188413 10.1097/TP.0000000000002440

[64] Park S, Kang SJ, Lee JW et al. Association between early post-transplant hypertension or related antihypertensive use and prognosis of kidney transplant recipients: a nationwide observational study. J Nephrol 2021;34:1457–65. 10.1007/s40620-021-01143-6 34487334

[65] Bae S, Ahn JB, Joseph C et al. Statins in kidney transplant recipients: usage, all-cause mortality, and interactions with maintenance immunosuppressive agents. J Am Soc Nephrol 2023;34:1069–77. 10.1681/ASN.0000000000000112 36890643 PMC10278772

[66] Holdaas H, Fellström B, Cole E et al. Long-term cardiac outcomes in renal transplant recipients receiving fluvastatin: the ALERT extension study. Am J Transplant 2005;5:2929–36. 10.1111/j.1600-6143.2005.01105.x 16303007

[67] Holdaas H, Fellström B, Jardine AG et al. Effect of fluvastatin on cardiac outcomes in renal transplant recipients: a multicentre, randomised, placebo-controlled trial. Lancet 2003;361:2024–31. 10.1016/S0140-6736(03)13638-0 12814712

[68] Bostom AG, Carpenter MA, Kusek JW et al. Homocysteine-lowering and cardiovascular disease outcomes in kidney transplant recipients: primary results from the Folic Acid for Vascular Outcome Reduction in Transplantation trial. Circulation 2011;123:1763–70. 10.1161/CIRCULATIONAHA.110.000588 21482964 PMC4887854

[69] Jardine AG, Fellström B, Logan JO et al. Cardiovascular risk and renal transplantation: post hoc analyses of the Assessment of Lescol in Renal Transplantation (ALERT) Study. Am J Kidney Dis 2005;46:529–36. 10.1053/j.ajkd.2005.05.014 16129216

[70] Carpenter MA, John A, Weir MR et al. BP, cardiovascular disease, and death in the Folic Acid for Vascular Outcome Reduction in Transplantation trial. J Am Soc Nephrol 2014;25:1554–62. 10.1681/ASN.2013040435 24627349 PMC4073424

[71] Carpenter MA, Weir MR, Adey DB et al. Inadequacy of cardiovascular risk factor management in chronic kidney transplantation—evidence from the FAVORIT study. Clin Transplant 2012;26:E438–46. 10.1111/j.1399-0012.2012.01676.x 22775763 PMC4388027

[72] Jardine AG, Gaston RS, Fellstrom BC et al. Prevention of cardiovascular disease in adult recipients of kidney transplants. Lancet 2011;378:1419–27. 10.1016/S0140-6736(11)61334-2 22000138

[73] van Dijk M, van Roon AM, Said MY et al. Long-term cardiovascular outcome of renal transplant recipients after early conversion to everolimus compared to calcineurin inhibition: results from the randomized controlled MECANO trial. Transpl Int 2018;31:1380–90. 10.1111/tri.13322 30106185

[74] Bredewold OW, Chan J, Svensson M et al. Cardiovascular risk following conversion to belatacept from a calcineurin inhibitor in kidney transplant recipients: a randomized clinical trial. Kidney Med 2023;5:100574. 10.1016/j.xkme.2022.100574 36593877 PMC9803830

[75] Rao NN, Stokes MB, Rajwani A et al. Effects of arteriovenous fistula ligation on cardiac structure and function in kidney transplant recipients. Circulation 2019;139:2809–18. 10.1161/CIRCULATIONAHA.118.038505 31045455

[76] Stoler ST, Chan M, Chadban SJ. Nutrition in the management of kidney transplant recipients. J Ren Nutr 2023;33:S67–72. 10.1053/j.jrn.2023.07.001 37482148

[77] Oellerich M, Sherwood K, Keown P et al. Liquid biopsies: donor-derived cell-free DNA for the detection of kidney allograft injury. Nat Rev Nephrol 2021;17:591–603. 10.1038/s41581-021-00428-0 34031575

[78] Loupy A, Aubert O, Orandi BJ et al. Prediction system for risk of allograft loss in patients receiving kidney transplants: international derivation and validation study. BMJ 2019;366:l4923. 10.1136/bmj.l4923 31530561 PMC6746192

[79] Divard G, Aubert O, Debiais-Deschamp C et al. Long-term outcomes after conversion to a belatacept-based immunosuppression in kidney transplant recipients. Clin J Am Soc Nephrol 2024;19:628–37. 10.2215/CJN.0000000000000411 38265815 PMC11108246

[80] Guideline Development Group. Clinical Practice Guideline on management of patients with diabetes and chronic kidney disease stage 3b or higher (eGFR <45 mL/min). Nephrol Dial Transplant 2015;30:ii1–142. 25940656 10.1093/ndt/gfv100

[81] Diker Cohen T, Polansky A, Bergman I et al. Safety of sodium-glucose cotransporter 2 inhibitors in kidney transplant recipients with diabetes mellitus. Diabetes Metab 2025;51:101627. 10.1016/j.diabet.2025.101627 39956453

[82] Lee SA, Verhoeff R, Hullekes F et al. SGLT2 inhibitors and GLP-1 receptor agonists in kidney transplantation: a systematic review and meta-analysis. Transplantation 2025. Online ahead of print. 10.1097/TP.0000000000005496 PMC13725813 40702593

[83] Oikonomaki D, Dounousi E, Duni A et al. Incretin based therapies and SGLT-2 inhibitors in kidney transplant recipients with diabetes: a systematic review and meta-analysis. Diabetes Res Clin Pract 2021;172:108604. 10.1016/j.diabres.2020.108604 33338553

[84] Mickiewicz A, Żegleń S, Kędzierska-Kapuza K et al. Lipid-lowering agents in solid organ transplant recipients. Nephrol Dial Transplant 2025;40:1659–71. 10.1093/ndt/gfaf104 40575919 PMC12394127

[85] Kohnle M, Pietruck F, Kribben A et al. Ezetimibe for the treatment of uncontrolled hypercholesterolemia in patients with high-dose statin therapy after renal transplantation. Am J Transplant 2006;6:205–8. 10.1111/j.1600-6143.2005.01132.x 16433776

[86] Ponticelli C, Arnaboldi L, Moroni G et al. Treatment of dyslipidemia in kidney transplantation. Expert Opin Drug Saf 2020;19:257–67. 10.1080/14740338.2020.1732921 32073914

[87] Amaro JM, Villanego F, Orellana CD et al. Management of dyslipidemia with evolocumab in kidney transplant recipients. Transplantation 2024;108:e74–6. 10.1097/TP.0000000000004942 38317278

[88] Sánchez Fructuoso AI, Bedia Raba A, Banegas Deras E et al. Sodium-glucose cotransporter-2 inhibitor therapy in kidney transplant patients with type 2 or post-transplant diabetes: an observational multicentre study. Clin Kidney J 2023;16:1022–34. 10.1093/ckj/sfad007 37260993 PMC10229265

[89] Lim J-H, Kwon S, Seo YJ et al. Cardioprotective effect of SGLT2 inhibitor in diabetic kidney transplant recipients: a multicenter propensity score matched study. Kidney Int Rep 2024;9:2474–83. 10.1016/j.ekir.2024.05.022 39156155 PMC11328785

[90] Sheu J-Y, Chang L-Y, Chen J-Y et al. The outcomes of SGLT-2 inhibitor utilization in diabetic kidney transplant recipients. Nat Commun 2024;15:10043. 10.1038/s41467-024-54171-8 39567483 PMC11579355

[91] Giri K, Dube GK. Is it time to expand the use of SGLT2 inhibitors in kidney transplant recipients? Kidney Int Rep 2025;10:660–2. 10.1016/j.ekir.2025.01.007 40225359 PMC11993677

[92] Bakker WM, Heerspink HJL, Berger SP et al. Rationale and design of the renal lifecycle trial assessing the effect of dapagliflozin on cardiorenal outcomes in severe chronic kidney disease. Nephrol Dial Transplant 2025;gfaf046. 10.1093/ndt/gfaf046 PMC12394133 40053493

[93] Sharif A, Chakkera H, de Vries APJ et al. International consensus on post-transplantation diabetes mellitus. Nephrol Dial Transplant 2024;39:531–49. 10.1093/ndt/gfad258 38171510 PMC11024828

[94] Girerd S, Jaisser F. Mineralocorticoid receptor antagonists in kidney transplantation: time to consider? Nephrol Dial Transplant 2018;33:2080–91. 10.1093/ndt/gfy065 29672718

[95] Mortensen LA, Jespersen B, Helligsoe ASL et al. Effect of spironolactone on kidney function in kidney transplant recipients (the SPIREN trial): a randomized placebo-controlled clinical trial. Clin J Am Soc Nephrol 2024;19:755–66. 10.2215/CJN.0000000000000439 38416033 PMC11168825

[96] Krisanapan P, Suppadungsuk S, Sanpawithayakul K et al. Safety and efficacy of glucagon-like peptide-1 receptor agonists among kidney transplant recipients: a systematic review and meta-analysis. Clin Kidney J 2024;17:sfae018. 10.1093/ckj/sfae018 38410684 PMC10896177

[97] Orandi BJ, Chen Y, Li Y et al. GLP-1 receptor agonists in kidney transplant recipients with pre-existing diabetes: a retrospective cohort study. Lancet Diabetes Endocrinol 2025;13:374–83. 10.1016/S2213-8587(24)00371-1 40056927 PMC12171561

[98] Kidney Disease: Improving Global Outcomes (KDIGO) Transplant Work Group. KDIGO Clinical Practice Guideline for the Care of Kidney Transplant Recipients. Am J Transplant 2009;9:S1–155. 10.1111/j.1600-6143.2009.02834.x 19845597

[99] de Vries LV, Dobrowolski LC, van den Bosch JJON et al. Effects of dietary sodium restriction in kidney transplant recipients treated with renin-angiotensin-aldosterone system blockade: a randomized clinical trial. Am J Kidney Dis 2016;67:936–44. 10.1053/j.ajkd.2015.11.026 26803690

[100] Afsar B, Afsar RE, Caliskan Y et al. A holistic review of sodium intake in kidney transplant patients: more questions than answers. Transplant Rev (Orlando) 2024;38:100859. 10.1016/j.trre.2024.100859 38749098

[101] De Smet S, Van Craenenbroeck AH. Exercise training in patients after kidney transplantation. Clin Kidney J 2021;14:ii15–24. 10.1093/ckj/sfab022 33981416 PMC8101622

[102] Greenwood SA, Koufaki P, Mercer TH et al. Effect of exercise training on estimated GFR, vascular health, and cardiorespiratory fitness in patients with CKD: a pilot randomized controlled trial. Am J Kidney Dis 2015;65:425–34. 10.1053/j.ajkd.2014.07.015 25236582

[103] Rampersad C, Bau J, Orchanian-Cheff A et al. Impact of donor smoking history on kidney transplant recipient outcomes: a systematic review and meta-analysis. Transplant Rev (Orlando) 2024;38:100854. 10.1016/j.trre.2024.100854 38608414

[104] Osté MCJ, Gomes-Neto AW, Corpeleijn E et al. Dietary Approach to Stop Hypertension (DASH) diet and risk of renal function decline and all-cause mortality in renal transplant recipients. Am J Transplant 2018;18:2523–33. 29464830 10.1111/ajt.14707 PMC6175360

[105] Kluch M, Kurnatowska I, Matera K et al. Nutrition trends in patients over the long term after kidney transplantation. Transplant Proc 2020;52:2357–62. 10.1016/j.transproceed.2019.12.055 32571697

[106] Svensson M, Jardine A, Fellström B et al. Prevention of cardiovascular disease after renal transplantation. Curr Opin Organ Transplant 2012;17:393–400. 10.1097/MOT.0b013e3283560a3b 22790074

[107] Devine PA, Courtney AE, Maxwell AP. Cardiovascular risk in renal transplant recipients. J Nephrol 2019;32:389–99. 10.1007/s40620-018-0549-4 30406606 PMC6482292

[108] Wissing KM, Abramowicz D, Weekers L et al. Prospective randomized study of conversion from tacrolimus to cyclosporine A to improve glucose metabolism in patients with posttransplant diabetes mellitus after renal transplantation. Am J Transplant 2018;18:1726–34. 10.1111/ajt.14665 29337426

[109] Thomusch O, Wiesener M, Opgenoorth M et al. Rabbit-ATG or basiliximab induction for rapid steroid withdrawal after renal transplantation (Harmony): an open-label, multicentre, randomised controlled trial. Lancet 2016;388:3006–16. 10.1016/S0140-6736(16)32187-0 27871759

[110] Vincenti F, Charpentier B, Vanrenterghem Y et al. A phase III study of belatacept-based immunosuppression regimens versus cyclosporine in renal transplant recipients (BENEFIT study). Am J Transplant 2010;10:535–46. 10.1111/j.1600-6143.2009.03005.x 20415897

[111] Durrbach A, Pestana JM, Pearson T et al. A phase III study of belatacept versus cyclosporine in kidney transplants from extended criteria donors (BENEFIT-EXT study). Am J Transplant 2010;10:547–57. 10.1111/j.1600-6143.2010.03016.x 20415898

[112] EMPA-KIDNEY Collaborative Group, Herrington WG, Staplin N et al. Long-term effects of empagliflozin in patients with chronic kidney disease. N Engl J Med 2025;392:777–87. 10.1056/NEJMoa2409183 39453837 PMC7616743

[113] The EMPA-KIDNEY Collaborative Group, Herrington WG, Staplin N et al. Empagliflozin in patients with chronic kidney disease. N Engl J Med 2023;388:117–27. 10.1056/NEJMoa2204233 36331190 PMC7614055

[114] Heerspink HJL, Stefánsson BV, Correa-Rotter R et al. Dapagliflozin in patients with chronic kidney disease. N Engl J Med 2020;383:1436–46. 10.1056/NEJMoa2024816 32970396

[115] Perkovic V, Tuttle KR, Rossing P et al. Effects of semaglutide on chronic kidney disease in patients with type 2 diabetes. N Engl J Med 2024;391:109–21. 10.1056/NEJMoa2403347 38785209

[116] Wyld MLR, De La Mata NL, Masson P et al. Cardiac mortality in kidney transplant patients: a population-based cohort study 1988-2013 in Australia and New Zealand. Transplantation 2021;105:413–22. 10.1097/TP.0000000000003224 32168042

[117] Stocklassa T, Borchert-Mörlins B, Memaran N et al. Sex differences in subclinical cardiovascular organ damage after renal transplantation: a single-center cohort study. J Womens Health (Larchmt) 2021;30:1352–61. 10.1089/jwh.2020.8594 33211603

[118] Balafa O, Fernandez-Fernandez B, Ortiz A et al. Sex disparities in mortality and cardiovascular outcomes in chronic kidney disease. Clin Kidney J 2024;17:sfae044. 10.1093/ckj/sfae044 38638550 PMC11024840

[119] Ministrini S, Padró T. What is the role of sex and gender in the future of precision cardiology? Eur Heart J 2024;45:4252–3. 10.1093/eurheartj/ehae504 39254039

[120] Wenzl FA, Kraler S, Ambler G et al. Sex-specific evaluation and redevelopment of the GRACE score in non-ST-segment elevation acute coronary syndromes in populations from the UK and Switzerland: a multinational analysis with external cohort validation. Lancet 2022;400:744–56. 10.1016/S0140-6736(22)01483-0 36049493

[121] Pihlstrøm HK, Mjøen G, Mucha S et al. Genetic markers associated with long-term cardiovascular outcome in kidney transplant recipients. Am J Transplant 2019;19:1444–51. 10.1111/ajt.15191 30457209

[122] Kiberd B, Panek R. Cardiovascular outcomes in the outpatient kidney transplant clinic: the Framingham risk score revisited. Clin J Am Soc Nephrol 2008;3:822–8. 10.2215/CJN.00030108 18322053 PMC2386708

[123] Israni AK, Snyder JJ, Skeans MA et al. Predicting coronary heart disease after kidney transplantation: Patient Outcomes in Renal Transplantation (PORT) Study. Am J Transplant 2010;10:338–53. 10.1111/j.1600-6143.2009.02949.x 20415903

[124] Soveri I, Holme I, Holdaas H et al. A cardiovascular risk calculator for renal transplant recipients. Transplantation 2012;94:57–62. 10.1097/TP.0b013e3182516cdc 22683851

[125] Chukwu CA, Rao A, Middleton R et al. Post-transplant cardiovascular disease in kidney transplant recipients: incidence, risk factors, and outcomes in the era of modern immunosuppression. J Clin Med 2024;13:2734. 10.3390/jcm13102734 38792274 PMC11122649

[126] Colombijn JMT, Idema DL, van Beem S et al. Representation of patients with chronic kidney disease in clinical trials of cardiovascular disease medications: a systematic review. JAMA Netw Open 2024;7:e240427. 10.1001/jamanetworkopen.2024.0427 38451526 PMC10921252

[127] Lepeytre F, Dahhou M, Zhang X et al. Association of sex with risk of kidney graft failure differs by age. J Am Soc Nephrol 2017;28:3014–23. 10.1681/ASN.2016121380 28592422 PMC5619967

[128] Cheung AK, Chang TI, Cushman WC et al. Executive summary of the KDIGO 2021 Clinical Practice Guideline for the Management of Blood Pressure in Chronic Kidney Disease. Kidney Int 2021;99:559–69. 10.1016/j.kint.2020.10.026 33637203

[129] Drawz PE, Beddhu S, Bignall ONR et al. KDOQI US Commentary on the 2021 KDIGO Clinical Practice Guideline for the Management of Blood Pressure in CKD. Am J Kidney Dis 2022;79:311–27. 10.1053/j.ajkd.2021.09.013 35063302

[130] Mancia G, Kreutz R, Brunström M et al. 2023 ESH Guidelines for the management of arterial hypertension The Task Force for the management of arterial hypertension of the European Society of Hypertension: endorsed by the International Society of Hypertension (ISH) and the European Renal Association (ERA). J Hypertens 2023;41:1874–2071. 37345492 10.1097/HJH.0000000000003480

[131] McEvoy JW, McCarthy CP, Bruno RM et al. 2024 ESC guidelines for the management of elevated blood pressure and hypertension. Eur Heart J 2024;45:3912–4018. 10.1093/eurheartj/ehae178 39210715

[132] Baker RJ, Mark PB, Patel RK et al. Renal association clinical practice guideline in post-operative care in the kidney transplant recipient. BMC Nephrol 2017;18:174. 10.1186/s12882-017-0553-2 28571571 PMC5455080

[133] Whelton PK, Carey RM, Aronow WS et al. 2017 ACC/AHA/AAPA/ABC/ACPM/AGS/APhA/ASH/ASPC/NMA/PCNA guideline for the prevention, detection, evaluation, and management of high blood pressure in adults: a report of the American College of Cardiology/American Heart Association Task Force on Clinical Practice Guidelines. Hypertension 2018;71:e13–115. 29133356 10.1161/HYP.0000000000000065

[134] de Boer IH, Khunti K, Sadusky T et al. Diabetes management in chronic kidney disease: a consensus report by the American Diabetes Association (ADA) and Kidney Disease: Improving Global Outcomes (KDIGO). Diabetes Care 2022;45:3075–90. 10.2337/dci22-0027 36189689 PMC9870667

[135] Rossing P, Caramori ML, Chan JCN et al. Executive summary of the KDIGO 2022 Clinical Practice Guideline for Diabetes Management in Chronic Kidney Disease: an update based on rapidly emerging new evidence. Kidney Int 2022;102:990–9. 10.1016/j.kint.2022.06.013 36272755

[136] Mottl AK, Alicic R, Argyropoulos C et al. KDOQI US commentary on the KDIGO 2020 Clinical Practice Guideline for Diabetes Management in CKD. Am J Kidney Dis 2022;79:457–79. 10.1053/j.ajkd.2021.09.010 35144840 PMC9740752

[137] American Diabetes Association Professional Practice Committee. 9. Pharmacologic approaches to glycemic treatment: standards of care in diabetes-2025. Diabetes Care 2025;48:S181–206. 10.2337/dc25-S009 39651989 PMC11635045

[138] Wanner C, Tonelli M, Kidney Disease: Improving Global Outcomes Lipid Guideline Development Work Group Members. KDIGO Clinical Practice Guideline for Lipid Management in CKD: summary of recommendation statements and clinical approach to the patient. Kidney Int 2014;85:1303–9. 10.1038/ki.2014.31 24552851

[139] Sarnak MJ, Bloom R, Muntner P et al. KDOQI US commentary on the 2013 KDIGO Clinical Practice Guideline for Lipid Management in CKD. Am J Kidney Dis 2015;65:354–66. 10.1053/j.ajkd.2014.10.005 25465166

[140] Mach F, Baigent C, Catapano AL et al. 2019 ESC/EAS guidelines for the management of dyslipidaemias: lipid modification to reduce cardiovascular risk. Eur Heart J 2020;41:111–88. 10.1093/eurheartj/ehz455 31504418

